# Plasma and Intracellular Antiretroviral Concentrations in HIV-Infected Patients under Short Cycles of Antiretroviral Therapy

**DOI:** 10.1155/2014/724958

**Published:** 2014-11-09

**Authors:** Laura Zehnacker, Emuri Abe, Dominique Mathez, Jean-Claude Alvarez, Jacques Leibowitch, Stéphane Azoulay

**Affiliations:** ^1^Institut de Chimie de Nice UMR 7272, Université Nice Sophia Antipolis, Parc Valrose, 06108 Nice Cedex 2, France; ^2^Laboratory of Pharmacology-Toxicology, AP-HP, Hôpital Raymond Poincaré, 104 Boulevard Raymond Poincaré, 92380 Garches, France; ^3^Immunology and Virology Unit, AP-HP, Hôpital Raymond Poincaré, 104 Boulevard Raymond Poincaré, 92380 Garches, France

## Abstract

Study of plasma and intracellular concentrations of atazanavir, lopinavir, nevirapine, and efavirenz was conducted on 48 patients under short cycles of antiretroviral therapy. Intracellular concentrations (IC) were still measurable for all drugs after 85 h or 110 h drug intake despite the absence of drug in plasma for atazanavir and lopinavir. A linear relationship between plasma and intracellular efavirenz was observed. Further studies to fully understand the impact of IC in the intermittent antiviral treatment are required.

## 1. Introduction

Despite new approved anti-HIV drugs and more effective combinations, uninterrupted antiretroviral therapy is still considered to be required to avoid treatment failure. However strict adherence to HIV treatment might be difficult to achieve regarding side effects or personal environment.

Leibowitch and coworkers have previously published a pilot study that evaluated the efficacy of intermittent antiviral treatment administered to HIV-infected patients under stepwise reductions in weekly medication [1]. During this study, forty-eight patients were invited to reduce their antiviral medication to 5 consecutive days per week (d/wk); after having made sure that viremia was still under control (<50 copies/mL), antiviral drugs were cut to 4 consecutive days per week. Of the 48, 39 then reduced medicines further to 3 d/wk, and 12 of those eventually undertook a 2 d/wk schedule. Interestingly, no major HIV-related clinical event was reported and CD4+ T-cell counts and percentages readily increased over the last value noted under the 7 d treatment course. Viral failure was documented in 6 of the 48 patients (4 under a 3 d/wk regimen, 2 under a 2 d/wk regimen). Nevertheless, no pharmacological data have been reported so far about these patients.

We undertook a retrospective analysis on both plasma and peripheral blood monoclear cells (PBMCs) which have been collected during the study. Plasma and intracellular concentrations (IC) were investigated for two protease inhibitors (PI), atazanavir (ATV) and lopinavir (LPV), and two nonnucleoside reverse transcriptase inhibitors (NNRTI), efavirenz (EFZ) and nevirapine (NVP).

## 2. Materials and Methods

All patients were selected on the basis of both their volunteering to follow an uncharted course of anti-HIV treatment and their adherence to repeated monitoring with the approval of the Raymond Poincaré Hospital Ethics Review Committee on Infectious Diseases. An individualized letter acknowledging the off-label, nonvalidated status of these exploratory prescriptions at their onset was personally addressed to and signed by each individual patient. Baseline characteristics of the patients and virological and immunological outcomes have been reported previously [1]. The majority of patients (91.1%) were on an emtricitabine (FTC)/tenofovir disoproxil fumarate (TDF) regimen combined with EFV (400 mg or 600 mg) or a ritonavir-boosted PI: ATZ (300 mg) or LPV (400, 600 or 800 mg) once a day, whereas patients taking NVP (400 mg) were on a didanosine (DDI)/FTC/TDF regimen daily. During the course of the pilot study done by Leibowitch and co., patients were monitored for plasma concentration regularly. Samples were collected in lithium heparin tubes (7 mL). Plasma obtained after centrifugation at 1400 g for 10 min at +4°C and PBMCs subsequently isolated were stored at −20°C until analysis. For the purpose of this study, only sample done at least two months after the modification of the treatment was taken into account to avoid transition state data.

All IC were measured by immunoassays we previously developed and PBMCs treated as previously described [2–5]. Briefly, PBMCs were isolated by using cell preparation tubes (4 mL Vacutainer CPT tubes; Becton Dickinson, Le pont de Claix, France). Aliquots of PBMCs were washed three times in ice-cold phosphate-buffered saline, centrifuged (2,000 ×g for 5 min at 4°C), and extracted in 1 mL of a methanol-H_2_O mixture (90/10; vol/vol). The number of PBMCs was determined following cell lysis by using a validated biochemical test which relies on an established DNA-based fluorescence signal [6]. The concentration is therefore expressed as the amount per 10^6^ cells. In order to compare intracellular and plasma concentrations, IC were converted into the amount per volume, based on the approximation that the PBMCs volume is 0.4 pL [7]. Plasma assays were done using a validated liquid chromatography method coupled to ion trap mass spectrometry detection with electrospray ionization interface. A basic liquid-liquid extraction with* ter*-butyl-methyl-ether was carried out using efavirenz-d_4_ and amprenavir-d_4_ as internal standards. Calibration curves were linear for all compounds in the 100–10000 ng/mL range. The limits of detection were 0.50 ng/mL for LPV and ATV and 50 ng/mL for NVP and EFZ. Only values in the calibration range were considered for ratio calculation. The intra- and interassay precisions evaluated at 400, 1500, and 8000 ng/mL were all <15% and the intra- and interassay accuracies were in the 93.5–106% range at the three concentrations.

Statistical analysis was performed using GraphPad Prism 5.03 computer software (GraphPad Software Inc., CA).

## 3. Results and Discussions

PI and NNRTI target viral enzyme into the infected cells. There is still debate whether intracellular concentrations could be a useful parameter for efficacy or toxicity prediction. However relatively few data are available on intracellular concentrations [8]. Intracellular concentrations may be an important determinant of antiviral activity, and the pharmacokinetics of intracellular drug accumulation is likely to impact upon efficacy and toxicity. Moreover an attempt to understand, in a pharmacological point of view, the control of viral load in patients under short cycles of antiretroviral therapy is needed. 48 patients were included in the study and viral failure was documented in 6 of the 48 patients. In total, more than 180 samples were collected. For the purpose of our analysis, results were divided in several groups regarding drugs, dosing, and time of collections since the last drug intake. However, the present study has two inherent limitations. First, no pharmacokinetic data could be calculated as samples at different time for the same patient were not collected consecutively to one drug intake. Second, the number of samples was, in some cases, limited and interpretations might be taken with caution.

Table 1 reports IC and intracellular-to-plasma concentration (IPC) ratios of all analyzed drugs. For LPV, IC are 274.7, 1571, and 537.1 fmol per 10^6^ cells for 400 mg, 600 mg, and 800 mg, respectively, 13 h after intake. Despite apparent difference in the median values, no statistical difference using one-way ANOVA followed by Tukey-Kramer test was noticed. The median IPC ratios are 0.49, 0.30, and 0.21, respectively. IC are 39.1 and 511.3 fmol per 10^6^ cells for 400 mg and 600 mg, respectively, 85 h after intake. In all cases, the IPC ratio is lower than previously reported [9]. Several factors influence intracellular drug concentrations such as plasma protein binding (altering the free fraction of drug), physiochemical properties (such as lipophilicity, degree of ionization), and cellular influx/efflux active transport [10, 11]. Multidrug transport proteins, such as P-glycoprotein (P-gp) or MRP1, function as a protective barrier to potential toxic agents lowering the intracellular concentration of a broad range of chemically unrelated substrates, a phenomenon known as multidrug resistance. Strong interindividual variations of their expression have often been reported and could account for the difference in reported data. Moreover drug-drug interactions may have great influence of multidrug transport proteins [12]. Altogether these factors could make interpretation of data complicated.

For ATV, IC are 1685 and 76.4 fmol per 10^6^ cells for 300 mg 13 h and 85 after intake, respectively. A high IPC ratio (3.04) is observed at 13 h; however no plasma concentration was detected at 85 h despite significant residual IC. So far, no clinical data has been published for ATV IC.

**Figure 1:**
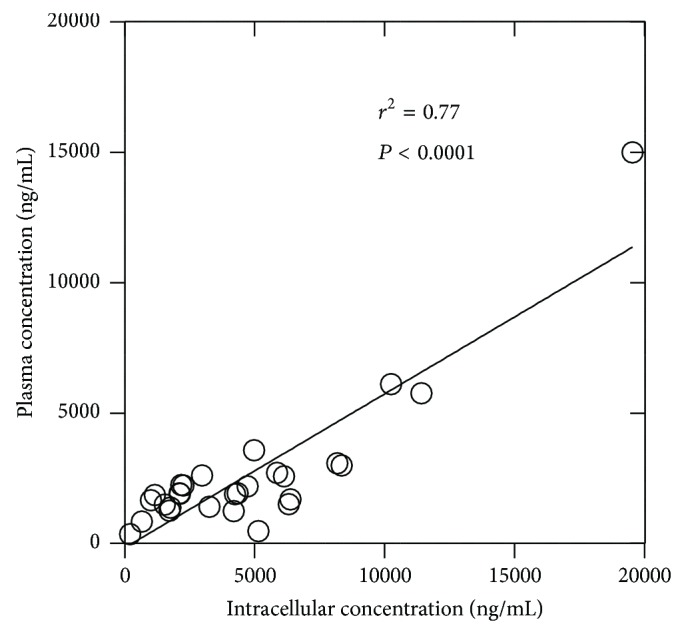
Relationship between intracellular and plasma concentrations for EFV.

For EFZ 400 mg, IC are 3081, 1131, 10250, and 547 fmol per 10^6^ cells for 400 mg 13 h, 60 h, 85 h, and 110 h after intake, respectively. The IPC ratios are 1.40, 1.84, 1.92, and 0.83, respectively. For EFZ 600 mg, IC are 3762, 2064, and 3707 fmol per 10^6^ cells for 600 mg 13 h, 60 h, and 85 h after intake, respectively. The IPC ratios are 1.40, 1.84, 1.92, and 0.83, respectively. As previously noticed [13], there is an accumulation into cells, except for 110 h, which remains constant over time. This observation is in accordance with a good linear relationship between IC and plasma concentration (observed at 13 h for EFZ 600 mg (Figure 1); *r* ^2^ = 0.77, *P* < 0.0001; for statistical validity only these data (*n* = 28) were analyzed). A relationship was already observed in another study [13], and in EFV case suggests that plasma EFV concentrations may be good surrogate markers for IC EFV.

For NVP, IC are 40.4, 69.5, and 7.2 fmol per 10^6^ cells for 400 mg 13 h, 85 h, and 110 h after intake, respectively. The IPC ratios are 0.006, 0.064, and 0.049. IC are very low compared to other drugs and in particular EFV, another NNRTI, and there is no intracellular accumulation which is in accordance with previous results [14]. However no correlation between IC and plasma concentration was observed.

It is noteworthy that for all drugs IC are still quantifiable after 85 h or 110 h drug intake even for PI when no plasma concentration was detected which may be due to shorter half-life for PI (5 h average [15, 16]) than EFV and NVP (25 h and 50 h, respectively, [17]). Moreover EFV maintains constant accumulation into cells. The fact that EFV is more lipophilic than NVP [18] could partially explain the great difference in terms of IC between the two compounds. However, the efficacy of NVP at such low IC level should be further explored. Despite an IPC ratio constant over the studied interval, the concentration for EFZ 400 mg and NVP 400 mg at 85 h tends to increase. No clear explanation could be done even if complex drug interactions could not be excluded but results should be confirmed on a larger number of patients. Measured intracellular concentrations for the patients who were in virological failure (1 EFZ 400 mg, 1 LPV 400 mg, 2 LPV 600 mg, and 1 NVP 400 mg) were all in the interquartile range but in the longer length of treatment interruption, that is, on 2 days or 3 days per week treatment. As already mentioned [1], three causes of failure could be identified after analysis ((1) nonadherence, (2)  reductions of the daily dose because of side-effects, and (3)  inappropriate daily dosage). In 5 of the 6 patients whose virus escaped under intermittent treatment, one single new mutation emerged within the HIV reverse transcriptase as a 184I amino acid change in 3 patients and a 103N amino acid change in 1 other patient. The fifth patient had had an additional 65R mutation in his previously highly mutated virus (carrying amino acid changes at positions 74V, 115F, 184V, 100I, and 103N in the RT genome). No genotype was available for the sixth failing patient, because his viral load under escape remained <200 copies/mL. For these 6 patients, reassignment to a 7-day per week regimen, with a new combination, led to HIV levels under control within 2 months.

In conclusion, this study provides complementary observations about rather limited IC data. A prospective designed clinical trial is needed for further warranty before considering short weekly cycles of antiretroviral medicines as an alternative and these results absolutely do not support that nonstrict adherence may equally be effective as current established treatment. The presence of intracellular drugs after several days of intake is a very interesting observation suggesting quite longer half-life than in plasma and may be one of the parameters explaining such viral control. Understanding of the mechanism of action should take into account intracellular concentrations. A better comprehension of intracellular pharmacology could improve long-term therapy.

## Figures and Tables

**Table 1:** Intracellular concentrations and intracellular/plasma concentration ratios.

	Time (h)^a^	Median (range) fmol per 10^6^ cells^b^	*n*	Intracellular/plasma concentration ratios^c^	*n*
ATV 300 mg	13	1685 [559.3–4085]	10	3.04 [1.94–3.23]	7
	85	76.4 [63.9–87.7]	3	nc^b^	

LPV 400 mg	13	274.7 [31.2–1241]	7	0.49 [0.11–0.68]	4
	85	39.1 [32–95.4]	3	nc^b^	

LPV 600 mg	13	1571 [807.2–3505]	4	0.30 [0.22–2.20]	3
	85	511.3 [61.34–659.4]	3	nc^b^	

LPV 800 mg	13	537.1 [247.9–922.9]	6	0.21 [0.13–0.72]	6

EFZ 400 mg	13	3081 [1385–9940]	10	1.4 [0.68–4.14]	9
	60	1131 [932.3–3514]	3	1.84 [1.21–2.77]	3
	85	10250 [628.9–20285]	5	1.92 [0.86–2.77]	4
	110	547 [263.7–3666]	5	0.83 [0.54–1.7]	4

EFZ 600 mg	13	3762 [2230–6490]	55	1.39 [1.02–2.31]	27
	60	2064 [1572–2631]	5	2.15 [1.79–3.13]	5
	85	3707 [1939–4521]	7	2.27 [0.95–4.42]	7

NVP 400 mg	13	40.4 [22.5–132.4]	25	0.0055 [0.003–0.0175]	20
	85	69.5 [30.8–140.3]	7	0.064 [0.008–0.103]	7
	110	7.2 [5.8–104]	3	0.049 [0.013–0.188]	3

^a^Time after intake.

^b^Quantitative variables are expressed as medians and interquartile ranges [*Q*1; *Q*3].

^c^Not calculated: no plasma concentration detected.
